# Authorial and institutional stratification in open access publishing: the case of global health research

**DOI:** 10.7717/peerj.4269

**Published:** 2018-02-19

**Authors:** Kyle Siler, Stefanie Haustein, Elise Smith, Vincent Larivière, Juan Pablo Alperin

**Affiliations:** 1 Innovation Studies Group, Copernicus Institute of Sustainable Development, Utrecht University, Utrecht, Netherlands; 2 School of Information Studies, University of Ottawa, Ottawa, ON, Canada; 3 Centre Interuniversitaire de Recherche sur la Science et la Technologie (CIRST), University of Québec at Montreal, Montreal, QC, Canada; 4 École de bibliothéconomie et des sciences de l’information, University of Montreal, Montreal, QC, Canada; 5 Canadian Institute for Studies in Publishing, Simon Fraser University, Vancouver, BC, Canada

**Keywords:** Open access, Publishing economics, Libraries, Global health, Stratification, Article processing charges, Universities

## Abstract

Using a database of recent articles published in the field of Global Health research, we examine institutional sources of stratification in publishing access outcomes. Traditionally, the focus on inequality in scientific publishing has focused on prestige hierarchies in established print journals. This project examines stratification in contemporary publishing with a particular focus on subscription vs. various Open Access (OA) publishing options. Findings show that authors working at lower-ranked universities are more likely to publish in closed/paywalled outlets, and less likely to choose outlets that involve some sort of Article Processing Charge (APCs; gold or hybrid OA). We also analyze institutional differences and stratification in the APC costs paid in various journals. Authors affiliated with higher-ranked institutions, as well as hospitals and non-profit organizations pay relatively higher APCs for gold and hybrid OA publications. Results suggest that authors affiliated with high-ranked universities and well-funded institutions tend to have more resources to choose pay options with publishing. Our research suggests new professional hierarchies developing in contemporary publishing, where various OA publishing options are becoming increasingly prominent. Just as there is stratification in institutional representation *between* different types of publishing access, there is also inequality *within* access types.

## Introduction

Publication in peer-reviewed journals legitimates and propagates academic research, while also underpinning professional and scientific reward structures for authors. Academic journals serve as conduits for new information and research, and also convey signals of academic status. For better or worse, scientific journals often function as quality signals for readers who may lack the interest and/or ability to thoroughly appraise the merit of an individual article, and thus play a pivotal role in the scientific reward system. As per McLuhan’s (1964) famous dictum, often in academic publishing, “the medium is the message.”

The accessibility and ubiquity of digitized knowledge on the Internet has changed scientific publishing in recent decades. Scientific research is now primarily disseminated online, even when published in traditional paywalled/subscription journals. The low cost and ease of information dissemination online has called the necessity of traditional publishing models into question. Although the traditional business model of for-profit scientific publishing funded via university subscriptions has proven remarkably resilient and profitable in the Internet age (Cookson, 2015), Open Access (OA) publishing options have emerged, offering benefits of improved public access and potentially lower publishing costs. Various forms of OA publishing challenge and/or complement traditional subscription-based business models of academic publishing. OA publishing has been—and has potential to continue to be—a *disruptive innovation* (Christensen, 1997), altering the markets and social hierarchies of the prevailing scientific publishing system.

OA journals publish scholarly research online, enabling free access for anyone with an internet connection (Suber, 2012). Publishing costs in gold OA journals often entail Article Processing Charges (APCs) assessed to authors publishing in a given journal. Notably, this shifts financial burdens from the demand side (libraries, readers) to the supply side (authors, researchers) of the market. In turn, OA publishing is not necessarily a panacea for financial challenges in contemporary publishing. Subscription journals create access problems in that not every institution can afford increasingly expensive subscriptions to all journals. In contrast, APCs run the danger of pricing out academics from being able to publish their work, especially those in developing countries or affiliated with less wealthy universities. Economic exclusion shifts from reading to publishing, raising the ethical question of which type of exclusion is less pernicious. The dual funding pressures of maintaining journal subscriptions at rising prices that exceed inflation (Association of Research Libraries, 2011), while also underwriting costs for APCs and/or running OA journals are particularly acute for less-wealthy universities. Researchers working at less-wealthy universities are being squeezed by escalating subscription costs for published journals, while also being expected to pay APCs for publishing in OA journals. Many institutions lack the resources to seriously devote funding to OA publishing, in part because library resources are being drained by subscription journals. *PLOS One* co-founder Michael Eisen (2013) warned, “[t]he APC model has serious problems for researchers without grant funding or from poor institutions.” (also see Moros, Halvorsen & Orton, 2016) Even when journals institute APC waivers for researchers without funding or from low to medium income countries, waivers are not always available to all who need them (Peterson, 2017), and put researchers with the least resources in the uncomfortable position of having to ask for assistance.

As a complement to other access types, green OA (often referred to as self-archiving) is an increasingly prominent means for disseminating academic work. While gold OA entails free access to final published typeset articles, green OA involves publishing articles before (i.e., preprints) or after (i.e., postprints) they may have undergone peer review, but without copy and layout editing (see Björk et al., 2014). In fields such as mathematics, physics and computer science, green OA has been well established through a strong pre-print culture in large part due to the prominent arXiv online repository (established in 1991). The arXiv model eventually diffused into economics, where the Social Science Research Network (SSRN) and Research Papers in Economics (RepEc) emerged as important publishing platforms. By the mid-2010’s, the success of these green OA repositories inspired establishment of additional repositories in biology (BioRxiv), chemistry (ChemRXiv), engineering (engrXiv), geosciences (EarthArXiv), humanities (Humanities Commons), law (LawArXiv), psychology (PsyArXiv) and social sciences (SocArXiv). Additionally, several universities established their own green institutional OA repositories. Green OA may be an effective compromise to funding and accessibility problems in publishing. Online repositories can host publicly accessible versions of published research, while the traditional publishing system remains intact, publishing formatted ‘final’ versions of articles in paywalled subscription-based journals. Since 2003, several universities and funding institutions have mandated Open Access, either through gold or green OA. Firm institutional policies mandating that researchers make published work publicly accessible have been found to make a positive difference in Open Access participation (Gargouri et al., 2012; Swan et al., 2015). However, the efficacy of such policies has been limited in many cases due to inflexible embargo periods imposed by leading publishers, inconsistent enforcement of OA mandates by relevant scientific stakeholders and low participation of researchers (Basken, 2016; Vincent-Lamarre et al., 2016; Schultz, 2017). ^1^ 1For example, Kaiser (2017: 1346) chronicled widespread skepticism—if not apathy—towards green OA in biology: “Although bioRxiv has grown rapidly, the more than 1,200 preprints deposited in it in August still represented just 1.3% of the 93,000 papers added to PubMed, the NIH-run database of biomedical abstracts, during that month.”

The ‘Hybrid’ publishing model occupies a niche bridging the traditional subscription-based publishing business model and newer APC-based OA models. Hybrid journals provide the opportunity to make articles in otherwise ‘closed’ subscription journals freely accessible for a fee, publishing those articles alongside regular paywalled articles, whose authors did not or could not pay the hybrid fee. However, this model has been widely criticized as exploitative because publishers ‘double-dip’, simultaneously deriving revenue from both APCs *and* journal subscriptions (Pinfield, Salter & Bath, 2016; Boyes & Kingsley, 2016). Further, publishers are able to strategically set high APC levels at to exploit wealthier, price-insensitive authors to maximize revenue, while OA articles remain rare enough within hybrid journals to justify continuing to charge full price for subscriptions. In journals offering such options, hybrid articles were relatively rare; only 3.8% of articles from 2011–2013 (Laakso & Björk, 2016), although the total number of hybrid journals and articles has steadily increased through 2016 (Björk, 2017). Well-meaning edicts from scientific stakeholders requiring OA publishing can underpin inelastic demand for hybrid publishing, which also enables publishers to set high APC price points in such journals. Perhaps unsurprisingly, APCs for hybrid journals have been found to be relatively higher than gold (APC) OA journals without hybrid options (Solomon & Björk, 2016; Jahn & Tullney, 2016; Haustein et al., 2016).

The costs and benefits of the various types of OA in the scholarly publishing ecosystem raise questions of whether access and adoption of different publishing types are equally spread throughout science. The traditional scientific publication system—with paywalled ‘closed’ print-based journals—is characterized by significant status hierarchies among established journals in most fields. This raises questions of whether—and if so, how—stratification between authors and institutions have developed in new OA publishing landscapes. Authors and institutions can differ in their interest and financial wherewithal to cover APC charges. These charges may especially onerous for scholars at universities and colleges with library budgets already stretched to cover escalating subscription costs for traditional print journals. Likewise, costlier OA options may be more accessible to authors affiliated with wealthier scientific institutions, such as elite universities, think tanks, research institutes and national funding organizations. Recent surveys of faculty found that knowledge of OA is slowly diffusing in academia. However, academics remain generally skeptical of the value of most OA publications for tenure and promotion, and continue to harbor concerns about the potential for inequities caused by cost-prohibitive APCs (Park & Qin, 2007; Meadows, 2012; Gaines, 2015; Peekhaus & Proferes, 2015; Nicholas et al., 2017). Another recent survey of 50,000 researchers revealed support for OA publishing, but also that APCs deter submissions to OA journals (Vogel, 2011). Further, APCs have varying effects on the incentives and choices of different researchers (Cotton, 2013).

Awareness of various OA alternatives (i.e., gold, green or hybrid publishing), as well as the importance placed on making research publicly accessible may also vary between authors and institutions. New innovations—such as Open Access publishing—are often adopted at different stages by people and institutions of different status levels (Bass, 1969; Rogers, 2003). Particularly with the diffusion and adoption of disruptive innovations, higher-status actors tend to hold advantages of access to greater resources, as well as the ability to legitimate and confer status upon previously unestablished innovations (Menzel, 1960). In other scenarios, marginal or semi-peripheral status may be conducive to innovation and early adoption, due to less investment in the status quo (McLaughlin, 2001; cf. Phillips & Zuckerman (2001) on middle-status conformity).

This article examines publishing choices made by scholars in different types of institutions working in the field of global health research. The multidisciplinary academic field of Global Health Research (GHR) can be related to numerous other fields including public health, international health and world health (Koplan et al., 2009). Such research includes diversity of stakeholders including researchers, health care institutions, university institutions, non-governmental organizations (NGOs), and community representatives in specific communities (Zarowsky, 2011). Have publishing hierarchies emerged in the new publishing ecology, which includes niches for numerous types of OA? If so, what does this new stratified publishing system look like?

## Methods

This study is based on a set of articles published from 2010–2014 and indexed under the Medical Heading Subject Identifier (MeSH) “Global Health” in PubMed, identified by Haustein et al. (2016) and Smith et al. (2017). This search yielded 3,336 Global Health research articles published in 909 different journals. In this article, we defined access categories at the paper level. These access categories are reported in Table 1 (also see Smith et al., 2017). In order to identify institutional addresses for all authors, the set of 3,336 PubMed documents was reduced to 2,067 documents published in journals indexed in the Web of Science with at least one institutional address. A final filter was applied, limiting the dataset to publications classified by the Web of Science as “Articles”, yielding a dataset of 1,352 articles. A limitation of our methodology is that we cannot directly observe how articles were solicited and/or whether a full or partial APC waiver was granted to the author(s) for each particular article. We used the Web of Science categorization of “Articles” as a reputable third-party vantage point to identify significant published works *most likely* to have involved an APC prior to publication. We expect that for most or all of the articles in our dataset, APCs influenced the publishing choices and experiences of authors, either through the transfer of money and/or perceptions of the official “sticker price” for publishing in the journal.

**Table 1:** Types and definitions of open access publishing.

Category	Definition
‘Free’ gold OA	Article published in a journal that does not charge authors an APC; the article is freely available to all readers immediately.
APC Gold OA	Article published in a journal that charges publishing authors an APC; the article is freely available to all readers immediately.
Delayed OA	Article published in a subscription-based journal; the article is freely available to all readers after an initial closed/paywalled period; also known as a moving paywall.
‘Hybrid’ publication	Article published in subscription-based journal; the article is freely available immediately to all readers if the publishing author pays an APC; hybrid articles are published alongside paywalled articles whose authors did not pay the APC.
‘Closed’ subscription-only publication	Article published in a subscription-based journal that does not offer the APCs; article is never freely available.
Green OA	Article is made freely available, irrespective of where it is published, by posting a copy in a subject or institutional repository or on another website.

The OA status of articles and associated APCs (converted to USD) were labeled based on journal and article-level information obtained from PubMed, the Directory of Open Access Journals, Ulrich’s Periodicals Directory as well as journal lists from Elsevier, Sage, Springer, Taylor and Francis and Wiley-Blackwell, as well as journal websites. Green OA was determined for non-gold and non-hybrid articles with Google searches for green OA archived versions of articles published in subscription journals (also see Smith et al., 2017: 3–5). Our access category definitions are based on the perspective of prospective readers—whether employed at universities or the general public—attempting to access scientific articles.

This study examines the relationship between scientific publishing options and institutional affiliations of authors. Institutional affiliations were coded for all first and last authors of every given article. While authorship order may represent varying professional relationships and work roles in different research articles, institutional affiliations for both first and last authors were analyzed, as they are the two authorship positions most likely to convey centrality, prestige and importance in the medical sciences (Azoulay, Graff Zivin & Wang, 2010: 563; Hundley, Teijlingen & Simkhada, 2013; West et al., 2013; Larivière et al., 2016). According to common authorship conventions, first author often conveys primary authorship, while the last position often entails supervision, Principal Investigator (PI) status and/or lab ownership (Dance, 2012; Pierson, 2014). In turn, first and/or last authors are deemed most likely to be responsible to (a) influence publishing choices and (b) to pay potential APCs. Based on the first listed affiliation for the first and last authors of each article, authors were assigned to the affiliations private company, government, hospital, non-profit organization, non-university research institute, scientific professional association or university. The first and second authors (KS and SH) independently coded institutional affiliations, resolving any discrepancies collaboratively. Although professional affiliations are sometimes complex, especially in cases where authors report multiple affiliations, we assumed the first listed affiliation is primary or most important for authors. All address information found in the Web of Science was cleaned and checked manually. Universities were also disaggregated by status. Universities and colleges were classified according to the 2017 *Times Higher Education* (*THE*) rankings, which ranked 981 institutions. We grouped universities into status-based categories: institutions ranked in the *THE* Top 50 and those with lower rankings (or were unranked). While we acknowledge the limitations of university rankings in general and the *THE* ranking in particular (e.g., see Ioannidis et al., 2007), they provide a rough indication of a university’s academic status, which also tends to be associated with financial resources (Espeland & Sauder, 2016).

## Results

Global health articles in our dataset were published using several different closed and open access publishing models (see Table 2).

**Table 2:** Number and percentage of papers, by access category (*N* = 1352).

Access category	Frequency	Percentage
Delayed OA Article	14	1.0
Gold OA Article (APC journal)	197	14.6
Gold OA Article (non-APC journal)	84	6.2
Green OA Article	461	34.1
Hybrid Article	122	9.0
Closed/Toll Access Article	474	35.1

The most common access category is the ‘closed’ or toll access option—35.1% of all papers—where articles are paywalled and readers must access them through a subscription or by paying a fee. Since all green OA papers were also published in toll access journals, this means that 69.2% of publications in our dataset were published in journals where the final, typeset version of the paper is paywalled (see Haustein et al., 2016; Smith et al., 2017). Roughly one-third of articles in our dataset were published in a green OA repository. Various gold OA alternatives have emerged as popular options for research dissemination. Roughly 30% of articles were published in various gold alternatives, including hybrid publishing. The 9% of hybrid articles in our dataset is relatively high, given industry-wide estimates of 4% (Laakso & Björk, 2016; Piwowar et al., 2017), and an additional estimate of 3% in health research (Piwowar et al., 2017). There is variability in the prevalence of hybrid OA in different disciplines although hybrid OA is relatively popular in global health research. This is indicative of a field that is interested both in public accessibility and traditional academic legitimacy, as well as being prone to having funding requirements for making research freely available. Delayed OA was relatively uncommon, accounting for only one percent of total publications.

As would be expected from a wide-ranging interdisciplinary field like global health publishing, authors came from a variety of institution types (Table 3). Unsurprisingly, universities are the most common type affiliation for both first and last authors, accounting for 61–64% of total affiliations. Highly-ranked universities are significantly overrepresented among authors in the corpus. Top 50-ranked universities alone account for roughly one-quarter of all publications. In this case, resources and prestige of larger, wealthier universities appear to be conducive to disproportionate prominence and productivity.

**Table 3:** Number and percentage of papers, by affiliation of first and last authors (*N* = 1,352).

Institution type	First author		Last author	
	Frequency	Percentage	Frequency	Percentage
Private Company	34	2.5	36	2.7
Government	80	5.9	84	6.2
Hospital	142	10.5	147	10.9
Non-Profit	149	11.0	166	12.3
Research Institute	72	5.3	75	5.6
Scientific Association	15	1.1	24	1.8
University—Rank 1–25	231	17.1	227	16.8
University—Rank 26–50	104	7.7	111	8.2
University—Rank 51–100	137	10.1	123	9.1
University—Rank 101–200	119	8.8	105	7.8
University—Rank 201–500	123	9.1	123	9.1
University—Rank 501+	49	3.6	43	3.2
University—Unranked	97	7.2	88	6.5

Non-profit organizations, governments and research institutes are also commonly represented among first and last authors in the global health corpus. Of course, many authors and academic departments have strong links to institutions outside of academia, while many hospitals and research institutes have formal and/or informal links to universities. Of 1,352 total articles in the database, 317 (23.4%) involved paying an APC. Table 4 reports quantiles of APC fees for journals in our dataset.

**Table 4:** Quantiles of APC costs in USD currency (*N* = 317).

Mean	Std. Dev.	Minimum	.25	Median	.75	Maximum
2,569.03	926.69	10.00	2,145.00	2,250.00	3,000.00	5,000.00

There is considerable heterogeneity in the costliness of APC-based OA publishing (also see Haustein et al., 2016). Charges ranged from $10 USD to $5,000 USD, with the median APC at $2,250 USD, slightly below the overall mean APC of $2,569 USD. In line with previous research, hybrid publications were significantly more expensive than other OA options. A comparison of means test of APCs revealed that hybrid APCs averaged $3,275 USD (SD = 681.75) while non-hybrid APCs averaged $2,139 USD (SD = 781.65) (*t* = 13.16).

Table 5 reports multivariate logistic regression models for the influence of institutional affiliation of authors on the propensity to publish manuscripts with different access types. The logistic command in Stata 14 was used with article access type (e.g., Gold APC, Hybrid) as the dependent variable to yield odds ratios. Both first and last authors of articles were analyzed. *University* is the omitted category from the models, meaning that results for each institutional category are relative to all universities and colleges in our dataset. There are no statistically significant associations between institution types on the likelihood of publishing with closed or gold (APC) access. Authors affiliated with governments and non-profit organizations are significantly more likely to publish in a free gold OA journal. There was also evidence that hospitals and non-profit organizations were less likely to use green OA to promote their work. In the case of government and non-profit authors, it appears that they prefer free gold OA to green OA. Hybrid publishing is particularly popular with authors affiliated with non-profit organizations, as well as those affiliated with private companies. Results also suggest that last authors affiliated with hospitals are relatively inclined to choose hybrid publishing

**Table 5:** Logistic regression of access type by authors’ institutional affiliation (odds ratios).

	Closed		Gold (APC)		Gold (Free)		Green		Hybrid	
	First author	Last author	First author	Last author	First author	Last author	First author	Last author	First author	Last author
Company	.641 (.254)	.800 (.295)	1.284 (.591)	1.112 (.509)	1.885 (1.178)	[Null]	.623 (.246)	.880 (.318)	3.225^***^ (1.430)	2.403 (1.120)
Government	.635 (.167)	.815 (.201)	.856 (.301)	.838 (.283)	3.437^***^ (1.206)	4.600^***^ (.1522)	.881 (.218)	.625 (.162)	.971 (.277)	1.092 (.454)
Hospital	1.062 (.199)	1.254 (.229)	.708 (.205)	.825 (.218)	.711 (.343)	1.058 (.447)	.856 (.164)	.681^*^ (.135)	1.177 (.249)	1.677 (.476)
Non-profit	.847 (.160)	.784 (.144)	1.511 (.342)	.849 (.211)	2.676^***^ (.795)	2.735^***^ (.810)	.531^***^ (.109)	.849 (.154)	1.658^**^ (.323)	1.740^*^ (.466)
Research Institute	1.203 (.301)	1.023 (.257)	1.198 (.397)	1.059 (.349)	1.146 (.617)	1.840 (.842)	.712 (.191)	.829 (.214)	1.200 (.341)	1.045 (.465)
Scientific Association	.892 (.492)	1.091 (.467)	.428 (.445)	.505 (.377)	[Null]	[Null]	1.514 (.791)	1.490 (.620)	.554 (.423)	.522 (.538)
University	[Omitted]	[Omitted]	[Omitted]	[Omitted]	[Omitted]	[Omitted]	[Omitted]	[Omitted]	[Omitted]	[Omitted]
Constant	.561^***^ (.040)	.550^***^ (.040)	.167^***^ (.016)	.180^***^ (.017)	.051^***^ (.008)	.047^***^ (.008)	.578^***^ (.041)	.568^***^ (.041)	.278^***^ (.023)	.083^***^ (.011)


**Notes.**

* *p* < .05.

** *p* < .01.

*** *p* < .001 (two-tailed tests).

Standard Errors are in parentheses.

Universities are not homogeneous institutions. Academic institutions vary in their location, mission, resources and reputation. Table 6 reports logistic regression results showing how affiliation with a Top 50-ranked university is associated with the likelihood of publishing with different access types. The status and/or resources generally associated with affiliation with a highly-ranked university appear to influence the publication choices of authors. Authors at Top 50-ranked universities were significantly less likely to publish in toll-access ‘closed’ journals. As a substitute, authors at Top 50-ranked universities were significantly more likely to choose gold APC OA journals to disseminate their work, but were less likely to choose Free gold OA journals. In turn, there appears to be institutional stratification in academic research, where authors at higher-status universities are more likely to opt-out of the traditional paywalled ‘closed’ publication access category, and choose gold APC OA. Further, academics at highly ranked institutions were relatively more likely to prefer APC options for gold publishing vis-à-vis free outlets, suggesting that access to greater resources influences specific APC preferences and choices. There were no statistically significant status differences in the propensity to choose green and hybrid options, although positive coefficients suggest that if anything, Top 50-affiliated scientists may also tend to have greater knowledge of and/or access to green and hybrid publishing options.

**Table 6:** Logistic regression of access type by university status (odds ratios; universities only—omitted group: non-top 50 university).

	Closed		Gold (APC)		Gold (Free)		Green		Hybrid	
	First author	Last author	First author	Last author	First author	Last author	First author	Last author	First author	Last author
Top 50 University	.673^**^ (.100)	.626^**^ (.095)	1.222 (.241)	1.494^*^ (.291)	.412^*^ (.158)	.590 (.216)	1.294 (.187)	1.286 (.189)	1.327 (.347)	1.002 (.267)
Constant	.651^***^ (.058)	.662^***^ (.062)	.154^***^ (.020)	.150^***^ (.020)	.067^***^ (.012)	.057^***^ (.011)	.522^***^ (.048)	.511^***^ (.049)	.071^***^ (.012)	.083^***^ (.014)

**Notes.**

* *p* < .05.

** *p* < .01.

*** *p* < .001 (two-tailed tests).

Standard Errors are in parentheses.

Table 7 reports APC differences of the journals authors published in, by institution type. University status is further disaggregated to show that relevant status boundary for APCs appears to be at the Top 500 university level. There is a noticeable drop-off in average APCs paid by universities in ranked above 500 in the *THE* rankings, and those that were not ranked at all. Non-profit organizations and hospitals also have mean APC costs noticeably above the mean level in our dataset. This may raise questions regarding whether paying relatively high APCs for publications are the best use of funds earmarked for charity or public health.

**Table 7:** Mean APCs paid for open access publications by institutional affiliation—Gold APC or hybrid only (Mean: 2569.03; *N* = 317).

	First author		Last author	
Institution type	Mean APC	*N*	Mean APC	*N*
Company	2,777.36	13	2,534.17	12
Government	2,596.56	16	2,562.06	17
Hospital	2,826.57	35	2,794.73	37
Non-profit	2,854.98	47	2,932.89	43
Research Institute	2,314.86	18	2,492.22	18
Scientific Association	2,150.00	2	1,933.33	3
University—Rank 1–25	2,692.70	56	2,599.50	59
University—Rank 26–50	2,369.64	26	2,490.71	29
University—Rank 51–100	2,448.55	30	2,465.20	33
University—Rank 101–200	2,524.09	22	2,439.00	20
University—Rank 201–500	2,380.02	30	2,322.05	30
University—Rank 501+	2,148.66	9	2,136.38	4
University: Unranked	1,963.46	13	2,191.67	12

## Discussion

Our results show that the current publishing landscape is a complex environment comprised of many types of dissemination and access options, as well as institutional stratification within and between those various access types. This reflects previous research (Björk & Solomon, 2012) showing considerable diversity in the ownership of OA journals and pricing principles used to support OA publishing. Our research also raises questions of what the specific forces and mechanisms are that underpin the propensities of academics to select different publishing access options. The case study of global health research suggests a number of trends in contemporary publishing.

Academics in high-status universities were more likely to publish in gold APC journals, and were less likely to choose ‘closed’ access options. This may be a counterintuitive finding, given that choosing newer OA journals can entail sacrificing prestige vis-à-vis high-ranked established print journals in the field. Could being an early adopter of OA publishing be a luxury enjoyed by wealthier, higher-status, more established academics? Or, could OA be attractive for early adopter researchers at lower-status institutions less invested in traditional hierarchies? Funding agencies may also play a role; do top universities receive more funding from institutions that mandate gold OA, and are willing and able to fund such a requirement? Higher-status academics and institutions are often professionally and intellectually conservative, in part because they benefit from the status quo (Bourdieu, 1988). However, high-status actors are also sometimes prone to being early adopters, since they tend to have the resources to invest in and legitimate new innovations (Menzel, 1960; Rogers, 2003); in this case, gold OA publishing and journals. Of course, like with traditional print journals, there is considerable heterogeneity in the quality and reputation of gold OA journals. While many reputable OA journals have emerged and now complement and/or compete with print-based journals, the existence of unscrupulous low-quality—or predatory—OA journals that will publish anything for APC revenue has been well documented (e.g., Bohannon, 2013).

While journal APCs do not directly influence the quality of submissions to a given publication, cost and overall journal quality tend to be linked. ‘Quality’ can entail a number of different journal attributes including reputational prestige, effective developmental and evaluative gatekeeping, well-organized and accessible metadata, aesthetically pleasing typesetting and a submission pool of high-quality articles. As Armstrong (2015: 10) argued, “[a] journal’s cost per article published will vary substantially, depending on how selective the journal is.” Research suggests a correlation between citations received by a journal and APC levels (Solomon & Björk, 2012a; West, Bergstrom & Bergstrom, 2014; Pinfield, Salter & Bath, 2016). This can further underpin incentives for OA journals to inflate impact factors (see Martin, 2016). In some cases, there may be a “chicken or egg?” dynamic with OA journals, where quality (whether real or perceived) allows for higher APCs, but at the same time, increased revenue enables curating a quality journal. Publishers take OA journal quality into account when pricing journals and authors are especially sensitive to the relationship between price and quality when deciding where to submit manuscripts (Björk & Solomon, 2015). Although oligopolistic forces influence journal pricing and can enrich publishers (Larivière, Haustein & Mongeon, 2015; Shulenburger, 2016), higher prices can also support quality control in academic research, optimal rejection rates and various costs in typesetting, promoting and curating quality research (Eisen, 2016). Cotton (2013) proffered an economic model by which journals set prices and publication lag times to maximize journal quality. Based on these trade-offs, different journals have different opportunities, incentives and niches when setting APCs. In turn, it may not be surprising that the ecology of OA journals is quite diverse.

The OA APC model changes journal incentives vis-à-vis the traditional subscription model. Rejecting articles becomes costly, both in terms of consuming journal resources, as well as forgoing opportunity costs of lost APCs (Jeon & Rochet, 2010; Gans, 2017: 56). Gold APC journals cover publication costs via fees paid by publishing authors. Rejected articles consume journal time and resources, but yield little or no revenue without an APC paid for publication. Journals need to build up substantial demand to support high rejection rates, where published authors are willing and able to pay higher APCs to cover the costs and forgone revenue associated with rejections for OA journals. Higher APCs can underpin exclusivity in a journal through limiting the submission pool via the financial constraints of researchers and/or by underwriting costs of high rejection rates.

Some OA journals are able to offer fee waivers to eligible authors, but this policy generally requires (1) sufficient revenue to support a fee waiver fund and (2) a sufficiently wealthy publishing demographic to render fee waiver requests and eligibility relatively uncommon. Some waivers are inconsistently applied; even scholars from low-GDP countries are sometimes deemed ineligible (Peterson, 2017). Further, within-country inequalities mean that even some researchers in developed countries still lack access to sufficient resources to participate. Institutions and research funders with OA mandates may be well-meaning, but can also cause inelastic demand for gold APC and hybrid publishing, which for-profit publishers can exploit with higher APCs. This diminishes the resources of institutions and scholars who can afford such fees, while excluding authors without the financial wherewithal to pay high APCs. In turn, journal APC cost and selectivity are both potential stratification mechanisms in contemporary scientific publishing.

If scientists at some institutions have fewer resources to support OA funding, it is not surprising that such authors are more likely to publish their work in less expensive or APC-free OA journals. Many scientists resort to personal funds to cover APCs, which also makes them much more likely to choose low-cost journals (Solomon & Björk, 2012b). Low-ranked, peripheral or downmarket scientific institutions often have different resources and incentives for researchers. While journal prices do not inherently imply scientific integrity or quality, Shamseer et al. (2017) identified low APCs as a common characteristic of predatory journals. In turn, the overrepresentation of authors in illegitimate, deceptive or ‘predatory’ journals from developing countries (Xia et al., 2015; Shen & Björk, 2015; cf. Pyne, 2017; Shamseer et al., 2017) may be more about incentives, resources and professional cultures in lower-status universities than internationalism. Lacking financial resources is an obvious mechanism that siphons scientists to lower-cost OA journals, which has implications for institutional and international stratification in scientific publishing.

In contrast to gold OA, green OA exhibited no stratification in adoption within academia. This suggests that knowledge of and access to green OA repositories has diffused widely in academia; or at least in the global health research community. Further, the relative lack of cost and selectivity constraints in most green OA repositories should have an equalizing effect on authors from a variety of institutions. Authors affiliated with research institutes, governments and non-profit organizations were relatively less likely to use green OA. In the case of non-profit organizations and governments, authors were substantially more likely to publish in free gold OA publications, suggesting a preference for gold in lieu of green. This may be explained by the fact that these organisations are less likely to have institutional repositories, and that a disciplinary repository—such as arXiv—is lacking in global health research. In turn, green OA appears to be more of an academic-based alternative, perhaps because academics have incentives to publish their work in traditional paywalled journals, while also finding ways to make their research publicly accessible. Awareness of green repositories also likely differs between academic and non-academic circles; institutional repositories tend to be affiliated with universities and green OA is less likely to be known as a publishing option outside of the ivory tower. Authors affiliated with governments and non-profit organizations may prioritize knowledge application over knowledge creation and basic academic research and thus have fewer reasons to emphasize academic publishing status hierarchies. In turn, such authors are more likely to choose free gold OA options, as opposed to going through paywalled journals. Authors affiliated with governments and non-profit organizations are significantly more likely to publish in a free gold OA journal. This may suggest such authors are concerned with fast and open dissemination of research findings, but are less concerned with academic eminence with their publication choices.

Instead of preferring free gold OA to green OA, authors at research institutes are more likely to choose hybrid publications. This suggests dual interests in academic status and public accessibility, while also suggesting that the research institutes in our database are relatively well-resourced to support hybrid publishing, which tends to be an expensive publishing option relative to gold OA journals (Jahn & Tullney, 2016; Pinfield, Salter & Bath, 2016). A recent survey found that scientists who published OA work in hybrid journals primarily chose to do so due to funding mandates for OA publishing (Nelson & Eggett, 2017). These well-meaning mandates may help underpin inelastic demand and higher APCs in hybrid journals, at significant costs to scientists and their benefactors (Shulenburger, 2016). Regardless, hybrid publishing may be an especially attractive proposition for authors and institutions who desire the cachet and rewards of publishing in a traditionally ‘closed’ journal, but who also have the resources, mission and desire to make their published work widely accessible.

When publishing in gold journals, lower-status authors were also more likely to prefer free gold OA options. Since it is expensive and organizationally difficult to simultaneously fund subscription and APC options for universities (Siler, 2017), it makes sense that smaller and less-wealthy institutions would be more likely to struggle to fully participate in both subscription and APC markets. All universities are burdened by the escalating journal costs, but money for OA publishing may be particularly scarce outside of wealthy institutions. If higher-status academics are more likely or able to derive the benefits from OA from publishing in such journals, as well as publishing in more expensive, more reputable OA journals, this is another factor underpinning the Matthew Effect in science (Merton, 1968), where higher-status scientists are able to parlay their existing status into further cumulative advantages. Our results suggest that contemporary publishing has stratified both *between* and *within* OA types. In turn, the OA world presents a different but analogous stratification order to those entrenched in more traditional publishing and scientific hierarchies.

An obvious direction for future work on OA adoption and stratification is to examine additional research communities and professional fields. Our study analyzed a specific field of research, providing evidence regarding how participation in the OA publication market varies according to the institutional status of authors. This analysis was exploratory, without specific hypothesis testing, which can be conducive to Type I errors. Global health research is a unique case study as a field with both academic and applied niches, as well as a commonly-invoked significant ethical imperative; seeking to promote and provide global health equity (Koplan et al., 2009). The widespread access to information enabled by OA publication has been promoted as a mechanism which can propagate increased equity and accessibility in health research (Siriwardhana, 2015; Chan, Kirsop & Arunachalam, 2005). If publishing hierarchies exist in global health, where equity considerations are at the core of the field’s culture, stratification may be even more pronounced in other scientific fields. Further, the use of PubMed and Web of Science can disproportionately exclude journals published in less-developed countries. Future work could also pay particular attention to OA access inequalities and the diffusion of scientific articles in low to middle income countries.

OA publishing hierarchies in academic communities with different (inter)disciplinary orientations, cultures and niches regarding publicizing research, and with members with access to different financial resources may differ than those in global health research. A larger-scale approach to analyze these phenomena across disciplines and professional fields may be possible with new services such as Unpaywall Data, which help to determine the various types of OA on the article level (e.g., Bosman & Kramer, 2018). Our research provides evidence to inform future research in this general line of inquiry. New axes of stratification are emerging in academic publishing, adding to the already complex tapestry of inequality in science.

##  Supplemental Information

10.7717/peerj.4269/supp-1 Supplemental Information 1 First author affiliation data

10.7717/peerj.4269/supp-2 Supplemental Information 2 Last author affiliation data

## Additional Information and Declarations

### Data Availability

The following information was supplied regarding data availability: The raw data has been provided as Supplemental Files.
